# Infection caused by *Klebsiella pneumoniae* ST11 in a patient after craniectomy

**DOI:** 10.1007/s12223-019-00718-y

**Published:** 2019-05-22

**Authors:** Dominika Ojdana, Jan Kochanowicz, Paweł Sacha, Anna Sieńko, Piotr Wieczorek, Piotr Majewski, Tomasz Hauschild, Zenon Mariak, Elżbieta Tryniszewska

**Affiliations:** 1 Department of Microbiological Diagnostics and Infectious Immunology, Medical University of Bialystok, 15a Waszyngtona Street, 15-269 Bialystok, Poland; 2 Department of Neurosurgery, University Hospital of Bialystok, 24a M. Sklodowskiej-Cure Street, 15-276 Bialystok, Poland; 3 Department of Microbiology, Institute of Biology, University of Bialystok, 1J Ciolkowskiego Street, 15-245 Bialystok, Poland

## Abstract

*Klebsiella pneumoniae* infections have always been an important problem in public health, but today, the increasing resistance of these bacteria to antibiotics due to β-lactamases production has renewed interest in *K. pneumoniae* infections. The aim of the study was to present a case of a neurosurgical patient with multidrug-resistant *K. pneumoniae* ST11 infection after craniectomy. Four *K. pneumoniae* isolates from various clinical materials of the patient undergone identification and susceptibility testing with the Vitek2 system. Tests for β-lactamases production were performed according to EUCAST guidelines. Strains were analyzed for *bla* genes responsible for β-lactamase production (*bla*_TEM_, *bla*_SHV_, *bla*_CTX-M_, *bla*_VIM_, *bla*_IMP_, *bla*_NDM_, *bla*_KPC_, *bla*_OXA-48_) using PCR. Moreover, the genetic relatedness of these isolates was determined by pulsed-field gel electrophoresis (PFGE) and multilocus sequence typing (MLST). All tested strain presented multidrug resistance. The highest susceptibility was observed for imipenem, meropenem, and ertapenem. The strain isolated from the nervous system was ESBL-positive with *bla*_SHV-11_, *bla*_TEM-1_, and *bla*_CTX-M-15_ genes. Additionally, the strain from urine was *bla*_KPC-3_-positive. Molecular typing revealed that all strains belonged to the same clone and identified two PFGE profiles. The analysis of MLST allelic profile showed that tested *K. pneumoniae* strains belonged to ST11. Identification of ST11 *K. pneumoniae* as etiological factor of infection unfavorably impacts on prognosis among neurosurgical patient after craniectomy.

## Introduction

*Klebsiella pneumoniae* is a Gram-negative bacterium that can be isolated from the digestive tract of healthy people and from the hospital environment (Maroncle et al. 2002). Although *K. pneumoniae* has been known as an opportunistic pathogen, its importance is constantly growing because of its increasing resistance to antibiotics due to β-lactamase including carbapenemase production (Pitout et al. 2015). Because infections caused by ST11 *K. pneumoniae* among patients after craniectomy are uncommon, we present a case of a 24-year-old patient with this infection (Li et al. 2016). The patient also presented a urinary tract infection caused by *K. pneumoniae* producing *K. pneumoniae* carbapenemase (KPC) enzymes responsible for resistance against carbapenems. Here, we discuss the importance of ST11 identification among *K. pneumoniae* on prognosis, diagnostic, and therapeutic difficulties among neurosurgical patient after craniectomy.

## Case presentation and discussion

On the third of February 2015, the patient was transported from stationary hospice to the Department of Neurosurgery, University Hospital of Bialystok, because of the general deterioration of his state of health and the visible convexity of his skull after craniectomy. On the day of admission, the patient was in serious condition, with low response to stimulus. Physical examination revealed body temperature 36.8 °C and blood pressure of 120/70 mmHg. Results of laboratory examination showed (reference range within parentheses) white blood cell (WBC) count of 12.28 × 10^3^/μL [4.00–10.00], red blood cell (RBC) count of 4.86 × 10^6^/μL [4.50–6.00], hematocrit (HCT) count of 40.3% [40.0–54.0], and platelet (PLT) count of 256 × 10^3^/μL [130–350]. The computer tomographic (CT) scan of the head revealed extensive brain edema and large subdural and, among hemispherical fluid spaces, ventricular enlargement concomitant with the level of thickened content. On the fourth of February 2015, the patient underwent surgery; during the procedure, fluid spaces (shown in the CT scan) proved to be vast purulent reservoirs, which were evacuated, drained, and collected for microbiological examination. The control CT scan of the head showed a significant decrease of fluid reservoirs but with persistent edema and hypotension, including in the brain stem. In the subsequent days of hospitalization at the Department of Neurosurgery, University Hospital of Bialystok, the patient presented a 5-day history of fever.

A course of intravenous (IV) antibiotics including amikacin (AM) (Biodacine, Polpharma, Poland) at a dose of 2 × 500 mg and ciprofloxacin (CIP) (Ciprofloxacin, Fresenius Kabi Deutschland GmbH, Germany) at a dose of 2 × 400 mg was administered on the third of February. The antibiotic therapy was modified based on results of susceptibility testing. Meropenem (MEM) (Meronem, AstraZeneca UK Ltd.) was administered on the sixth of February in the intravenous course at a dose of 3 × 2 g. Unfortunately, the patient did not improve clinically. On the tenth of February 2015, the patient’s death occurred with a background of cardiorespiratory failure. Postmortem examination was not performed.

A rectal swab, pus from fluid spaces, and a urine sample were obtained from the patient for analysis during his stay in the department. The initial material for the study was a rectal swab to determine colonization of the patient. Next, pus from fluid spaces was taken to examine changes in the existing infection. The next day, a urine sample was collected due to an episode of fever. The cultures of all samples were positive for *K. pneumoniae*, whereas, from the urine sample, two different *K. pneumoniae* isolates were obtained. The results of antibiotic susceptibility tests of all tested strains are presented in Table 1.

**Table 1:** Characteristics of tested *K. pneumoniae* isolates

Isolates ID	Date of result approval	Source	MIC (mg/L)															β-lactamase
			AMC	FEP	CAZ	CTX	CXM	IMP	MEM	ETP	AM	GN	TOB	COL	CIP	TZP	SXT	
2625	06 February 2015	Rectal swab	R ≥ 32	R 8	R ≥ 64	R ≥ 64	R ≥ 64	S ≤ 0.25	S ≤ 0.25	S ≤ 0.5	I 16	R ≥ 16	R ≥ 16	S 0.125	R ≥ 4	R ≥ 128	R ≥ 320	*bla*_SHV-11_, *bla*_TEM-1_, *bla*_CTX-M-15_
2691	06 February 2015	Pus from fluid spaces	R ≥ 32	R 8	R ≥ 64	R ≥ 64	R ≥ 64	S ≤ 0.25	S ≤ 0.25	S ≤ 0.5	I 16	R ≥ 16	R ≥ 16	R ≥ 16	R ≥ 4	R ≥ 128	R ≥ 320	*bla*_SHV-11_, *bla*_TEM-1_, *bla*_CTX-M-15_
2823-1	11 February 2015	Urine	R 16	R 16	R 16	R ≥ 64	R ≥ 64	S ≤ 0.25	S 0.025	S ≤ 0.5	R ≥ 64	R ≥ 16	R > 256	R 32	R ≥ 4	R 64	R ≥ 320	*bla*_SHV-11_, *bla*_TEM-1_, *bla*_CTX-M-15_
2823-2	11 February 2015	Urine	R ≥ 32	R ≥ 64	R ≥ 64	R ≥ 64	R ≥ 64	R ≥ 16	R > 35	R ≥ 8	R ≥ 64	R ≥ 16	R 4	R 8	R ≥ 4	R ≥ 128	R ≥ 320	*bla*_SHV-11_, *bla*_TEM-1_, *bla*_CTX-M-15_, *bla*_KPC-3_

*Abbreviations: MIC* minimum inhibitory concentration, *AMC* amoxicillin with clavulanic acid, *FEP* cefepime, *CAZ* ceftazidime, *CTX* cefotaxime, *CXM* cefuroxime, *IMP* imipenem, *MEM* meropenem, *ETP* ertapenem, *AM* amikacin, *GM* gentamicin, *TOB* tobramycin, *COL* colistin, *CIP* ciprofloxacin, *TZP* piperacillin with tazobactam, *SXT* trimethoprim with sulfamethoxazole, *R* resistant, *S* susceptible, *I* intermediate

A test for β-lactamase production was prepared and interpreted according to the European Committee on Antimicrobial Susceptibility Testing (EUCAST) guidelines (2017). The extended-spectrum β-lactamase (ESBL) detection test revealed enhanced zones of inhibition between discs with ceftazidime (CAZ) (30 μg) and discs with cefotaxime (CTX) (30 μg) toward amoxicillin with clavulanic acid (AMC) (10/20 μg) for isolates obtained from the rectal swab (2625), pus from fluid spaces (2691), and the first strain from the urine sample (2825-1). Additionally, for these strains, phenotypic tests for metallo-β-lactamase (MBL), OXA-48, and KPC were negative. For one strain, the combination disc test with meropenem (10 μg) and meropenem (10 μg) with boronic acid showed a difference in the size of the inhibition zone greater than 5 mm, and the inhibition zone for temocillin (30 μg) was 6 mm. The second isolate obtained from the urine sample (2825-2) was suspected of KPC β-lactamase or OXA-48 production. Additionally, that strain showed a positive result in the biochemical Carba NP test (Nordmann et al. 2012). The obtained results indicated carbapenem resistance mediated by β-lactamase production among the second strain isolated from the urine sample.

The tested strains were analyzed for the presence of resistance mechanisms against β-lactam antibiotics using polymerase chain reaction (PCR) amplifications for β-lactamase genes (*bla*_TEM_, *bla*_SHV_, *bla*_CTX-M_) and for *bla* genes responsible for carbapenemase production (*bla*_VIM_, *bla*_IMP_, *bla*_NDM_, *bla*_KPC_, *bla*_OXA-48_) with the use of primers as described previously (Ojdana et al. 2015). The results of PCRs and sequencing are presented in Table 1.

**Fig. 1:**
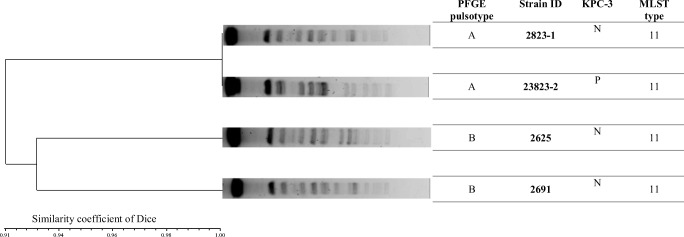
Comparison of PFGE pulsotypes produced by *Xba*I digestion and MLST of tested *K. pneumoniae* strains. Abbreviations: N: negative, P: positive

Analysis of chromosomal DNA restriction patterns by PFGE was carried out for determination of phylogenetic relatedness of tested *K. pneumoniae* isolates (Tenover et al. 1995). Molecular typing of four *K. pneumoniae* isolates revealed that all tested strains belonged to the same clone and identified two PFGE profiles (Fig. 1). Both urine isolates (2823-1, 2823-2) were classified as indistinguishable, whereas strains from the rectal swab (2625) and pus from fluid spaces (2691) presented close relatedness. The analysis of allelic profile (*rpoB*, *gapA*, *mdh*, *pgi*, *phoE*, *infB*, *tonB*) with the use of the *K. pneumoniae* MLST sequence type database (http://bigsdb.web.pasteur.fr/perl/bigsdb/bigsdb.pl?db=pubmlst_klebsiella_seqdef_public&page= sequenceQuery) showed that the tested *K. pneumoniae* strains belonged to the ST11 type.

The current spectrum of infection agents for post-neurosurgical infections, according to Yadegarynia et al. (2014), involves *Acinetobacter baumannii*, *K. pneumoniae*, methicillin-resistant *Staphylococcus aureus* (MRSA), *Staphylococcus saprophyticus*, *Pseudomonas aeruginosa*, methicillin-sensitive *S. aureus* (MSSA), and *Enterococcus faecalis*. Also, Kourbeti et al. (2015), in the study regarding the microbiological characteristics of infections among patients after craniotomy, revealed that Gram-negative pathogens including *Acinetobacter* spp., *Klebsiella* spp., *P. aeruginosa*, *Enterobacter cloacae*, and *Proteus mirabilis* presented 88% of the pathogens. Gram-negative rods including *Enterobacteriaceae*, followed by coagulase-negative staphylococci (CNS), *S. aureus*, and *Streptococci*, were also described as main etiological factors of surgical site infection after craniotomy by Korinek et al. (2006). However, according to Chiang et al. (2014), after evaluation of surgical site infections (SSIs) after craniotomy or craniectomy (CRAN), *S. aureus* was the most common etiological agent, whereas, among 104 analyzed cases, *K. pneumoniae* was the etiological factor of SSIs after CRAN among only three cases. In summary, we noticed that the actual epidemiological data presented varied contribution of individual pathogens among individual years and groups of patients presenting various factors predisposing to infections.

We present a case of a 24-year-old man with a history of craniectomy who developed multidrug-resistant *K. pneumoniae* ST11 infection after craniectomy. The strain isolated from the nervous system (2691) presented an ESBL resistance mechanism against β-lactam antibiotics, which significantly influenced the therapeutic options for patient treatment. Despite wide dissemination of ESBL enzymes among Gram-negative bacteria, the occurrence of ESBL-producing ST11 *K. pneumoniae* as an etiological factor of the neurological site infection is rare. According to Khan et al. (2014), among eight episodes of *K. pneumoniae* nosocomial post-neurosurgical infections with clinical presentation, one isolate was an ESBL producer. Yaita et al. (2012) presented six cases of post-trauma and/or postoperative ESBL-producing *K. pneumoniae* meningitis in the years 1990–2012. Moreover, Li et al. (2016), after the analysis of seventeen *K. pneumoniae* ST11 isolates during an outbreak in a Chinese teaching hospital, observed that only two isolates originated from a neurosurgical site.

*K. pneumoniae* ST11 belong to one of the main pathogenic clones of *K. pneumoniae* widespread in Asia, Latin America, the USA, and in European countries (Czech Republic, Spain, Greece, Switzerland) (Sun et al. 2015; Pitout et al. 2015). Additionally, *K. pneumoniae* ST11 are responsible for nosocomial infections of the urinary tract, bacteraemia, and infections of lower respiratory tract (Ko et al. 2010). Moreover, they are hypervirulent due to production of surface antigen (capsular polysaccharide), siderophores, and adherence factors (1 and type 3 fimbriae) (Zhan et al. 2017; Lee et al. 2018). Furthermore, they carry genes encoding antimicrobial resistance mechanisms which determine their multidrug resistance (Dong et al. 2018; Liu et al. 2018; Dsouza et al. 2017). In conclusion, ST11 identification among *K. pneumoniae* may unfavorably impact on prognosis, diagnostic, and therapeutic difficulties among neurosurgical patient after craniectomy.

In considering the origin of the ESBL-positive isolate in neurosite infection, we took into account the possibility of the endogenous transmission of the involved bacteria. According to publications, the gastrointestinal tract is an important reservoir of *Enterobacteriaceae* bacteria (Donskey 2004). The fact that an ESBL-positive *K. pneumoniae* strain from the rectal swab of a patient in the department was previously isolated may support that theory. Direct spread of ESBL-positive *K. pneumoniae* from the gastrointestinal tract was suspected. Additionally, the isolates from the rectal swab (2625) and pus from fluid spaces (2691) presented similar patterns of resistance to AMC, cefepime (FEP), CTX, CAZ, cefuroxime (CXM), piperacillin with tazobactam (TZP), AM, gentamicin (GM), tobramycin (TOB), CIP, and trimethoprim/sulfamethoxazole (SXT). The isolate from the rectal swab (2625) presented susceptibility to colistin (COL), whereas the strain from the pus from fluid spaces (2691) was resistant. Moreover, the PFGE analysis revealed these isolates presented close relatedness. O’Neill et al. (2006) demonstrated that preexisting external ventricular drains or ventriculoperitoneal shunts had been the entry sites for Gram-negative bacteria. Moreover, according to Sękowska and Gospodarek (2007), the use of vascular or urological catheters and assisted breathing predisposed patients to *K. pneumoniae* infections. These observations can indicate a possibility of the endogenous transmission of *K. pneumoniae* in our patient who required a central line, urinary catheter, and tracheostomy tube.

Moreover, in our study, we present the isolation of KPC-producing *K. pneumoniae* from our patient from a urine sample (2825-2). We could not identify the source or origin of the KPC-positive strain. The occurrence of strains resistant to carbapenems may have been the result of induction of resistance to antibiotics or an outcome of the strain acquisition by the patient from the hospital environment.

In light of the dangerous implications of the first possibility—the induction of carbapenem resistance to antibiotics—serious consideration is merited. Chia et al. (2010) investigated the mechanisms underlying carbapenem resistance. OmpK36 loss represents the major mechanism for the development of carbapenem-resistant *K. pneumoniae* (CRKP) in ESBL-producing isolates. Carbapenems have widely been used as the drug of choice to treat serious infections caused by *K. pneumoniae*-producing ESBL. Song et al. (2009) described in vivo development of resistance against carbapenem under meropenem selection pressure. The nature of the described resistance included OmpK36 loss during treatment with meropenem. From the epidemiologic history of our patient, we knew that CAZ and AM were previously administered to the patient, but these antibiotics are not known as carbapenem-resistance inducers.

From the patient’s history, we were aware of his stay in a stationary hospice. We could assume that the strain came from that environment. Over the last years, carbapenemase-producing *K. pneumoniae* (CPKP) strains have been detected not only among hospitalized patients but also among residents of long-term care facilities (Saegeman  et al. 2015). Our analyses indicated a significant degree of spread among microorganisms with resistance mechanisms against antibiotics such as ESBLs or even KPC enzymes (Ojdana et al. 2015). Considering the acquisition of a strain from the hospital environment, we determined the genetic relationship of isolated strains using the PFGE method. This analysis showed that all tested isolates presented genetic similarity. All tested strains were divided into two pulsotypes: strains obtained from the urine sample were indistinguishable, and the isolate from the rectal swab was closely related to the isolate from pus from fluid spaces. Obtained results may not have clearly represented the origin of the KPC-producing *K. pneumoniae* strain and further tests are required.

In summary, ST11 *K. pneumoniae* are hypervirulent and multidrug-resistant bacteria that may present diagnostic and therapeutic difficulties*.* Identification of ST11 *K. pneumoniae* as etiological factor of infection unfavorably impacts on prognosis among neurosurgical patient after craniectomy.
